# Is it time to talk about the end of social distancing? A joinpoint analysis of COVID-19 time series in Brazilian capitals

**DOI:** 10.1590/0037-8682-0469-2020

**Published:** 2020-09-21

**Authors:** Raphael Mendonça Guimarães, Mônica de Avelar Figueiredo Mafra Magalhães, Diego Ricardo Xavier, Raphael de Freitas Saldanha, Rafael de Castro Catão

**Affiliations:** 1 Fundação Oswaldo Cruz, Escola Politécnica de Saúde Joaquim Venâncio, Rio de Janeiro, RJ, Brasil.; 2 Fundação Oswaldo Cruz, Instituto de Comunicação e Informação em Ciência e Tecnologia, Rio de Janeiro, RJ, Brasil.; 3 Universidade Federal do Espírito Santo, Departamento de Geografia, Vitória, ES, Brasil.

**Keywords:** SARS-CoV-2, COVID-19, Social Distance, Time series, Brazil

## Abstract

**INTRODUCTION:**

Monitoring coronavirus disease (COVID-19)-related infections and deaths in Brazil is controversial, with increasing pressure to ease social distance measures. However, no evidence of a sustained, widespread fall in cases exists.

**METHODS:**

We used segmented (joinpoint) regression analysis to describe the behavior of COVID-19 infections in Brazilian capital cities.

**RESULTS:**

All capitals showed an exponential or a near-exponential increase in cases through May. A decline in reported cases was subsequently noted in 20 cities but was only significant for 8 (29.6%) and was followed in two by a renewed increase.

**CONCLUSIONS:**

Caution is warranted when considering the relaxation of restrictions.

In December 2019, China released the first report of coronavirus disease (COVID-19)^1^. Approximately one month later, World Health Organization (WHO) declared a public health emergency, and in March 2020 upgraded the status of the original disease to a pandemic^2^. At the end of June 2020, data showed that more than 10 million people were infected by severe acute respiratory syndrome coronavirus 2 (SARS-CoV-2) worldwide and more than 500,000 deaths^3^.

The Brazilian Ministry of Health confirmed that the first case of COVID-19 in Latin America occurred in Brazil in late February^4^. Less than a month later, Brazil declared a state of community transmission in the country. The Brazilian Ministry of Health adopted the WHO recommendations to close all public services and kept trade restricted to supermarkets, pharmacies, delivery restaurants, gas stations, and other critical services. Brazil’s structure as a federated republic creates relative autonomy for states and municipalities. Thus, social distancing and other COVID-19-related measures were adopted mostly at the state- and city-levels. Therefore, the level of social distancing varies nationwide^5^.

Almost four months since the first confirmed case in Brazil, the number of cases and deaths continues to increase. At the subnational level, some areas are still experiencing the initial trend of exponential growth, and some local health systems and funeral services have reached their threshold^6^. 

Brazil is rife with public disagreements about social distancing and the severity of the quarantine measures adopted by most state governors. The federal government faces an institutional crisis regarding the necessary steps to be taken to protect the population. Simultaneously, the accuracy of the criteria that define COVID-19 cases and deaths is being questioned in the parliament^7^.

Meanwhile, some states are planning to relax their quarantine rules despite the collapses experienced by some local health systems. The main argument fueling this decision is that a recent change in the transmission curve has been observed, with a tendency toward stability and decline. Thus, the objective of this study is to describe the behavior of the historical series of COVID-19 cases in Brazilian state capitals and identify changes in the trend over the course of the epidemic.

We performed the analysis using case notification data for Brazilian capitals, stratified as epidemiological week. Data were obtained from the Secretaries of Health of each of Brazil’s 26 states and the national capital of Brasília. To assess whether the trends changed over time, we used the Joinpoint segmented regression^8^ method. This statistical model identifies significant changes in a pattern over a period of time by assuming a trend between inflection points (“joinpoints”). A significant change between one point and the next is assumed to mark an inflection point and the beginning of a new regression trend. One of the advantages of this method is the ability to identify the number and location of changes in the trend, and to estimate the average percent change (APC) for each period defined between inflection points. Joinpoint regression models are particularly useful in evaluating continuity constraint at change points over time. Significant changes in trends are assessed using Monte Carlo approximate permutations to calculate the p value at each time point under the null hypothesis of no change in the trend. We maintained the overall asymptotic significance level to determine where to locate the joinpoints on the time scale through Bonferroni correction of the global alpha level^9^.

We used epidemiological weeks as the time unit, from the first 2020 epidemiological week to the 27^th^ epidemiological week (ending on July 4). We chose to perform the analysis using capitals rather than using federation units (states) to ensure data stability as well as to minimize the effect of inner state heterogeneity. To avoid autocorrelation between the dependent and independent terms of the regression equation, we used a “week-centered” variable as the time unit. Moreover, to guarantee the assumption of homoscedasticity, we used Poisson distribution parameters with robust variance. In this way, we adjusted the regression by considering the number of cases weekly as the dependent variable and the centered week as the independent variable. The selection of the number of inflection points was performed automatically by the software. We considered the level of significance at 5%.

All capital cities showed an exponential increase in new cases. To date, in most capitals the epidemiological week with the highest case count has been the 22^nd^ week (May 24th to May 30th). The full pattern of the growth curves, however, varied in each capital. A distinct drop was detected in 20 of the 27 cities (74.1%) and was significant in 8 (Belém, Maceió, Recife, Rio Branco, Rio de Janeiro, São Luís, São Paulo, and Vitória). However, in most cities, the speed (slope) of the decline was much lower than that of the increasing rate before the curve’s peak. We can check that looking at the curves (Supplementary material #1). Finally, it is essential to note that Belo Horizonte and Salvador experienced a renewed increase after a short period of decline (Table 1).

**TABLE 1:** Time trend in the occurrence of new cases of COVID-19 per epidemiological week in Brazilian capital cities, 2020

Capital	Period #1			Period #2			Period #3		
	EW	APC	p value	EW	APC	p value	EW	APC	p value
Aracaju	1 to 22	66.00	<0.001	22 to 27	-2.24	0.602			
Belo Horizonte	1 to 22	40.24	<0.001	22 to 25	-17.81	0.302	25 to 27	97.77	<0.001
Belém	1 to 20	65.46	<0.001	20 to 27	-13.60	<0.001			
Boa Vista	1 to 27	31.22	<0.001						
Brasília	1 to 27	24.03	<0.001						
Campo Grande	1 to 27	34.29	<0.001						
Cuiabá	1 to 27	26.07	<0.001						
Curitiba	1 to 27	26.95	<0.001						
Florianópolis	1 to 27	14.46	<0.001						
Fortaleza	1 to 19	53.38	<0.001	19 to 27	-11.71	0.001			
Goiânia	1 to 27	28.13	<0.001						
João Pessoa	1 to 22	66.91	<0.001	21 to 27	-6.34	0.083			
Macapá	1 to 27	22.22	<0.001						
Maceió	1 to 22	60.83	<0.001	22 to 27	-16.86	<0.001			
Manaus	1 to 20	47.58	<0.001	20 to 27	-14.52				
Natal	1 to 27	26.23	<0.001						
Palmas	1 to 27	23.26	<0.001						
Porto Alegre	1 to 27	18.79	<0.001						
Porto Velho	1 to 22	74.68	<0.001	22 to 27	-10.47	0.053			
Recife	1 to 19	54.75	<0.001	19 to 27	-16.56	<0.001			
Rio Branco	1 to 20	76.92	<0.001	20 to 27	-11.62	<0.001			
Rio de Janeiro	1 to 22	40.36	<0.001	22 to 27	-16.88	0.009			
Salvador	1 to 22	57.07	<0.001	22 to 25	-19.39	0.085	25 to 27	52.08	<0.001
São Luís	1 to 27	22.39	<0.001	22 to 27	-32.01	<0.001			
São Paulo	1 to 22	34.97	<0.001	22 to 27	-10.00	0.002			
Teresina	1 to 27	22.79	<0.001						
Vitória	1 to 22	55.70	<0.001	22 to 27	-10.76	<0.001			

**Source:** National System of Communicable Diseases (SINAN). **Legend: EW** - Epidemiological week; **APC** - Average weekly percent change.

The study of spatio-temporal patterns can help us understand the mechanisms of disease spread in a population, allowing the detection of behaviors in time and spatial clusters that represent characteristics related to dissemination^10^. Specifically, the use of segmented regression analysis enabled us to identify points in time when the trends changed significantly, generating quantitative hypotheses about changes in the behavior of the pandemic among Brazilian capitals. 

The rapid increase in COVID-19 clinical cases, characterized by the exponential pattern of the accumulated case curve, suggests that its transmissibility is high, with the basic reproductive number (R_0_) most likely between two and four^11^. Preventive strategies should aim to reduce this value, and the earlier and more rigorous the adoption of these measures, the more efficient they become^12^.

Understanding the stage of the epidemic we are currently in is essential for predicting scenarios^13^. In this regard, it is critical to mention that state governments and their health authorities are under increasing pressure to control the spread of COVID-19, seeking to assess the need for isolation measures and the flexibility of economic activities simultaneously. Therefore, an analysis of time-trend curves helps delineate the best window to identify a phase change in the epidemic. In countries that identified the pandemic early, the slowdown in growth rates was possible owing to the adoption of timely quarantine, rigorous social distancing, and isolation of the affected population^14^. Notably, the epidemic rate evolution in these countries shows two stages of slow down: the first occurs immediately after the adoption of lockdown measures, followed by a second shorter course about two weeks after implementation of these actions^15^. Although the 2-week period is related to the serial interval of the disease rather than a coincidence, the effects of relaxing or strengthening preventive measures are only reflected about two weeks later. Furthermore, even for the capitals with an observed change in the trend, caution is necessary as the changes are very recent, largely without statistical significance and with no evidence that they will be persistent over time.

The marked difference between the capitals may reflect differences in the measures adopted and the adherence of the population to those measures. The rate of decline in the curves is less than the rate of increase at the beginning of the epidemic. Any speculation regarding this decline and the use of the information to justify the flexibility and accelerated opening of public activities thus requires caution. The cases of Belo Horizonte and Salvador can be considered, wherein the rate of new infections declined, followed by an increasing rate higher than that in the first phase in short period. Finally, the decreasing trends in the capitals may be an indication of the epidemic’s migration to inland cities, The health service network will have to pay careful attention, as most likely, people who have contracted the infection will travel to the capitals for treatment, and without social distancing policies, thousands of susceptible people will be exposed to these cases.

Modeling is limited by the availability and quality of data, which in our case was the underreporting of new infections. Lack of tests is a barrier to establish the real number of people infected. The actual number of COVID-19 cases is undoubtedly much higher. Additionally, access to screening tests is different between the capitals^16^. The accuracy of case information depends on many factors such as the availability of screening tests and the speed with which health surveillance programs report suggested cases. Efforts to understand the dynamics of the disease to guide public policies should involve the analysis of existing information^17^.

Alfano & Erconalo^18^ reported that lockdowns were effective in reducing the number of new cases in the countries that implemented it, especially when considering a 10-20 day gap between the adoption of this strategy and the observed impact on incidence rates. However, some mathematical models suggest that this effect (reduction of case numbers) depends on the percentage of symptomatic cases that exists in the population^19^. Furthermore, evidence indicates that a stepwise implementation of measures that begins with social distancing in the epicenter of the city, followed by the province and the nation, would be practical and cost-effective without requiring a lockdown of the epicenter^20^. Nonetheless, to learn the real number of infections and test these assumptions, mass testing is required and this is a significant conundrum in Brazil.

The general recommendation is that the social distancing measures should be gradually eased in regions where the number of cases is few and an efficient public health system that can meet medium and high complexity medical demands such as intensive care units exists. Furthermore, before relaxing these guidelines, the country’s testing capacity needs to be substantially increased, considering Brazil’s continental dimensions and regional differences, including demographics, climate, urbanization, health care structure, and socioeconomic aspects. 

The joinpoint analysis only offers a description of the time series, and we did not intend to establish a firm conclusion on social distancing measures based solely on temporal changes. In fact, the results indicate that it is too early to make radical decisions on returning to pre-lockdown activities involving potentially large groups of people. Owing to numerous uncertainties, we consider it helpful to be cautious about returning to activities that would result in several people on the streets. Given the current situation, it would be wise to propose that the social distancing measure remain firm, despite their economic and social repercussions. Lastly, the current phase of the epidemic requires special attention to be paid to the needs of medium- and small-sized cities.
