# Supplementary material for: Is it time to talk about the end of social distancing? A joinpoint analysis of COVID-19 time series in Brazilian capitals
Source: Rev Soc Bras Med Trop. 2020 Sep 21;53:e20200469. doi: 10.1590/0037-8682-0469-2020 (PMC7508200; doi:10.1590/0037-8682-0469-2020)
Supplement: Supplementary file 1 [file 1678-9849-rsbmt-53-e20200469-suppl1.pdf]

Supplementary Material #1: Time series of new cases of COVID-19 according to epidemiological week in Brazilian capitals, 2020.

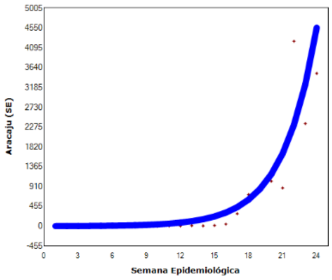

Aracaju

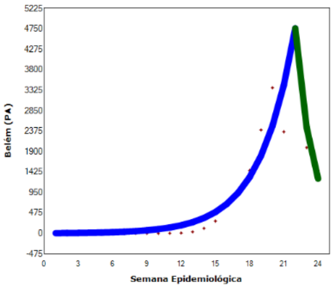

Belém

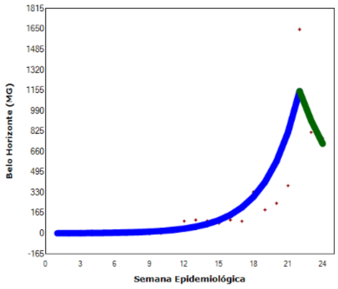

Belo Horizonte

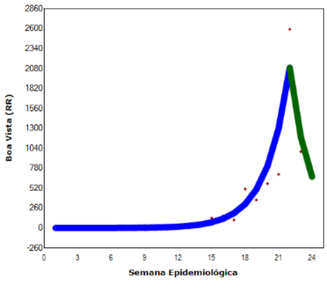

Boa Vista

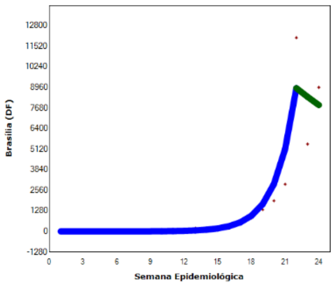

Brasília

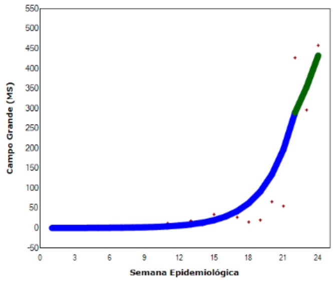

Campo Grande

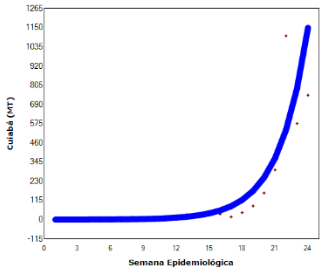

Cuiabá

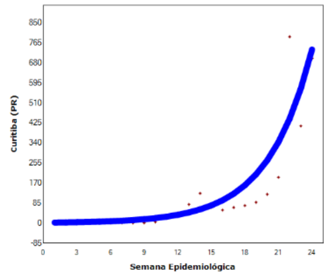

Curitiba

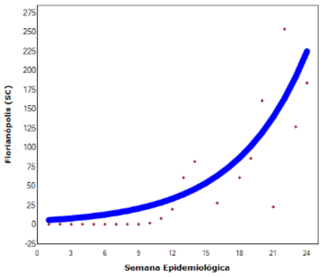

Florianópolis

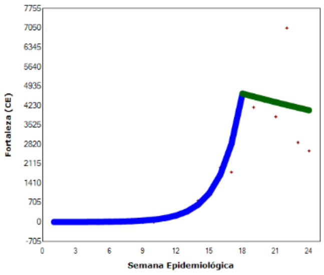

Fortaleza

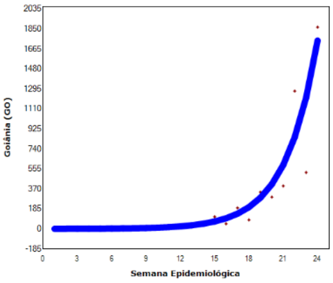

Goiânia

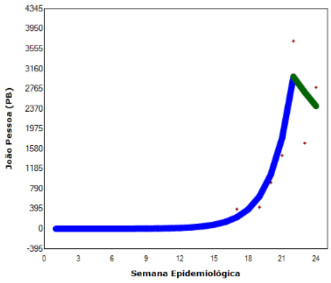

João Pessoa

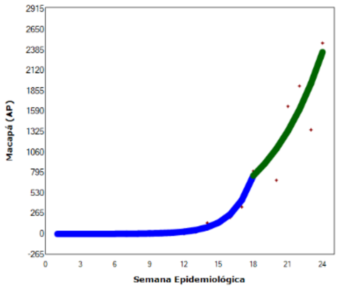

Macapá

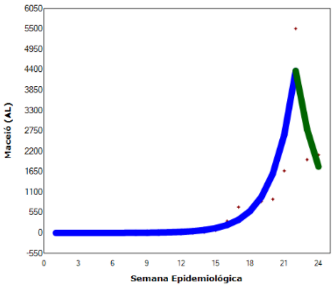

Maceió

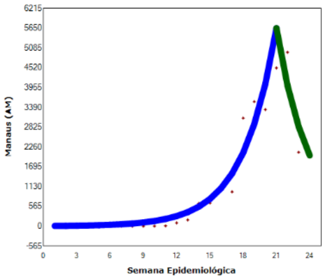

Manaus

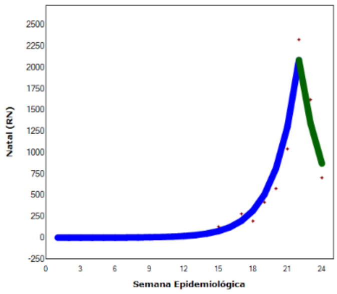

Natal

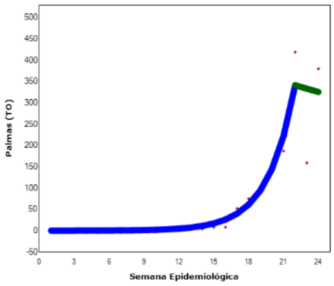

Palmas

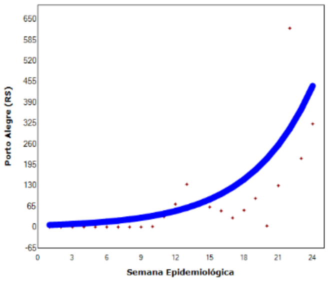

Porto Alegre

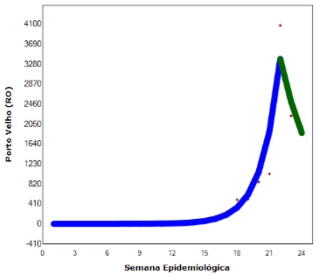

Porto Velho

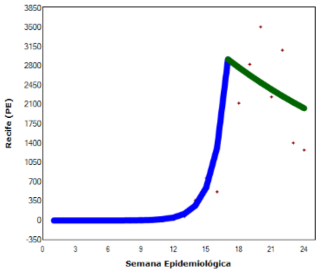

Recife

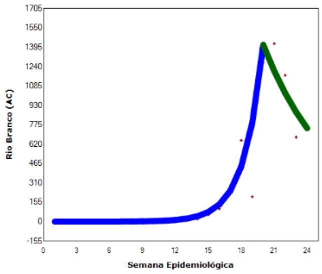

Rio Branco

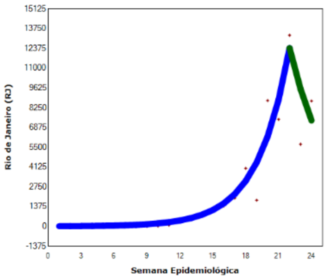

Rio de Janeiro

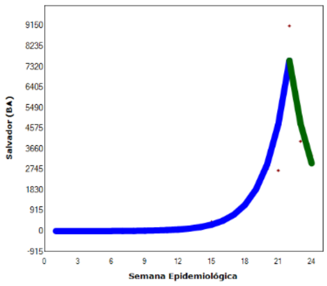

Salvador

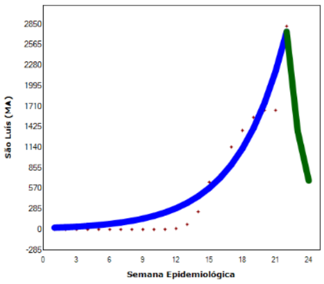

São Luís

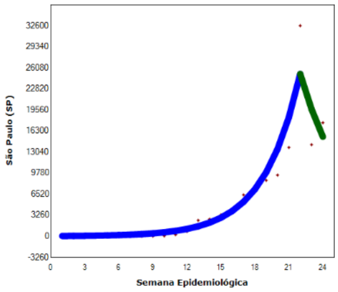

São Paulo

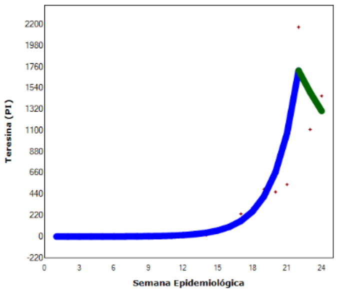

Teresina

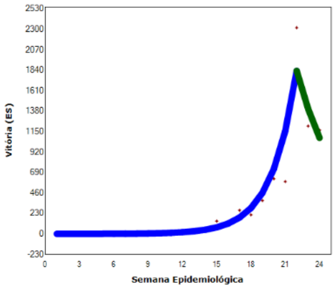

Vitória
